# Field testing of a household-scale onsite blackwater treatment system in Coimbatore, India

**DOI:** 10.1016/j.scitotenv.2020.136706

**Published:** 2020-04-15

**Authors:** Claire M. Welling, Sarani Sasidaran, Prateek Kachoria, Sarah Hennessy, Brendon J. Lynch, Stephanie Teleski, Hitendra Chaudhari, Katelyn L. Sellgren, Brian R. Stoner, Sonia Grego, Brian T. Hawkins

**Affiliations:** a Duke University Center for WaSH-AID and Department of Electrical and Computer Engineering, Durham, NC, USA; b RTI India, New Delhi 110 092, India; c RTI International, Research Triangle Park, NC, USA; d Triangle Environmental Health Initiative, Durham, NC, USA; e Biomass Controls, PBC, Durham, NC, USA

**Keywords:** WASH, Onsite sanitation, Blackwater reuse, Tamil Nadu, User testing

## Abstract

4.2 billion people live without access to safely managed sanitation services. This report describes the field testing of an onsite prototype system designed to treat blackwater from a single flush toilet and reuse of the treated effluent for flushing. The system passes wastewater through a solid-liquid separator followed by settling tanks and granular activated carbon columns into an electrochemical reactor that oxidizes chloride salts from urine to generate chlorine to remove pathogens. The objectives of the study were to verify the functionality of the system (previously demonstrated in the laboratory) under realistic use conditions, to identify maintenance requirements, and to make a preliminary assessment of the system's user acceptability. The prototype was installed in a women's workplace and residential toilet block in Coimbatore, India, and tested over a period of 10 months. The treated water met stringent disinfection threshold for both *E. coli* and helminth eggs and produced a clear, colorless effluent that met or nearly met local and international discharge standards for non-sewered sanitation systems. The effluent had an average chemical oxygen demand of 81 mg/L, total suspended solids of 11 mg/L, and reduction of total nitrogen by 65%. These tests determined the recommended service lifetimes and maintenance intervals for key system components including the electrochemical cell, granular activated carbon columns, and solid-liquid separator. User feedback regarding the use of treated blackwater as flush water was positive. These findings will inform the design and implementation of next-generation systems currently under development.

## Highlights

• 4.2 billion people worldwide lack access to safely managed sanitation.
• One approach to improving access is decentralized/onsite wastewater treatment.
• An onsite blackwater treatment system was field tested in Coimbatore, India.
• Functionality was demonstrated in the field.
• Failure modes and maintenance requirements were identified.

## Graphical abstract

**Figure:**
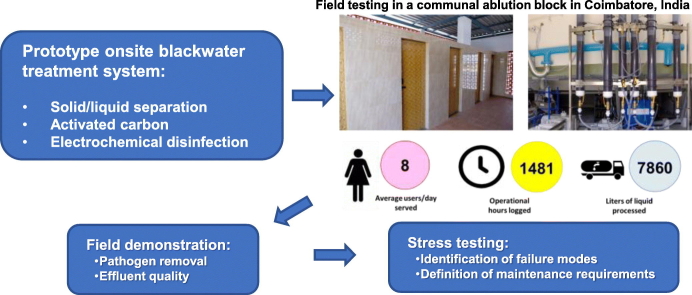

## 1 Introduction

A significant portion of the 4.2 billion people worldwide who live without access to safely managed sanitation services (UNICEF and WHO, 2019) reside in India. Despite the $1.5 billion investment from 2016 to 2017 of the nationwide sanitation campaign called Swachh Bharat, current infrastructure throughout all of India has the capacity to treat only 37.6% of the sewage generated (Government of India, 2017). As in many emerging countries, people in India largely depend on onsite sanitation systems such as septic tanks and pit latrines, both of which result in septage that is improperly disposed of, contaminating surface waters and groundwater (Krithika et al., 2017). This lack of adequate sanitation results in major economic losses through lost productive time, healthcare costs, and mortality. Improving sanitation and drinking water supply services could stimulate significant economic benefits (Hutton et al., 2007).

To address the global sanitation problem, the Bill & Melinda Gates Foundation announced the Reinvent the Toilet Challenge in 2011. The Challenge's goals for the reinvented toilet include treating wastewater onsite, achieving 100% pathogen removal, recovering energy, clean water, and nutrients, operating without a connection to clean water or sewer systems and economic sustainability (Bill and Melinda Gates Foundation, 2013). Several reinvented toilet systems have been developed under this program in response to the challenge (Cid et al., 2018; Hennigs et al., 2019; Yermán et al., 2015). Our team developed a system designed to treat waste from a single toilet that utilizes solid/liquid separation, where the liquid is settled through a series of gravity-fed settling tanks, run through granular activated carbon (GAC) columns for further treatment, and electrochemically treated for disinfection (Hawkins et al., 2017; Hawkins et al., 2019; Rogers et al., 2018; Sellgren et al., 2017). The system is designed to produce a pathogen-free effluent which can be reused as flush water. The solids are treated separately by a subsystem to be discussed in another report.

This paper describes the field testing of a prototype liquid treatment system in a residential/workplace setting in Coimbatore, India. A contemporaneous field test of this system was also conducted in Durban, South Africa (Sahondo et al., 2019). In this study, the objectives were: 1) to verify the functionality and durability of this system under daily usage over many months, 2) to identify maintenance requirements under realistic patterns of use, and 3) to make a preliminary assessment of the user acceptability of this technology. User-centered design is recognized as an essential guide to engineering development that, when implemented early in product development, can prevent potential downstream barriers to technology adoption (Morrison et al., 2018). Field testing with a representative population of intended users is therefore crucial to ensure harmonization of design and performance with their practices and preferences. For this study, the users (all women) had uninterrupted access to the toilet facilities 24 h/day, 7 days/week, and the quality of the liquid treatment process and the performance of system components were monitored comprehensively over a period of 10 months by an engineering team onsite.

## 2 Materials and methods

### 2.1 Test site and study design

**Fig. 1:**
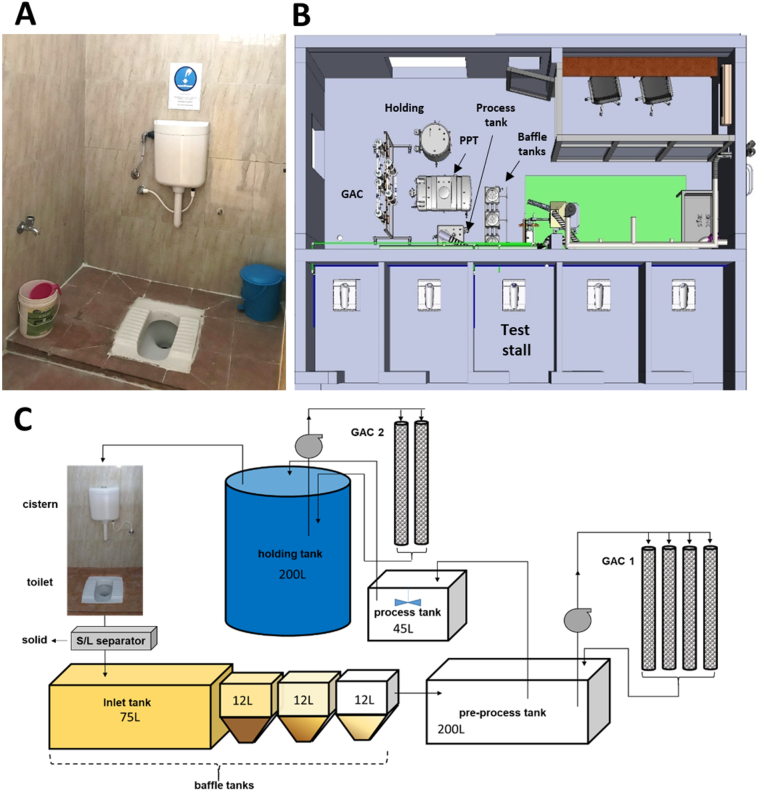
Overview of test site and prototype system. A: Toilet stall with squat plate, tap water line for wash water and flush water. B: Superstructure schematic of toilet stalls; one stall (the test stall) is connected to the prototype in a separate engineering room where blackwater is processed and samples are collected. C: Schematic of the prototype system: S/L separator: solid/liquid separator, PPT: pre-process tank, GAC1: first set of GAC columns, GAC2: second set of GAC columns.

The prototype was installed and tested at a privately-owned textile mill. The toilet block was used by women and featured 10 stalls (Fig. 1A), 10 separate showers, four taps for hand washing, and three clothes washing (laundry) stations. The block served an adjacent dormitory which housed 20 women working at the mill. The resident age typically ranged from 18 to 28 years with most women originating from the northern Indian state of Odisha. The toilet block was also available to approximately 50 non-resident women working at the mill. The onsite research staff included one male and one female engineer, the latter of whom could access the toilet area and spoke the local language (Tamil). Staff who could translate between Tamil and Odia (the language of many of the resident women) were available to facilitate communication with the users of the toilet block.

The prototype system was housed in an engineering area adjacent to the toilet block. The flush water and waste from one of the toilets was diverted to and processed by the prototype system (Fig. 1B). A valve was placed between the toilet and the prototype to enable diversion to the septic tank during system maintenance and downtime. All the other toilets were connected directly to septic with accessible connections in the engineering area. Each toilet stall consisted of a squat plate, a water faucet tap and a bucket for washing, as most people in India practice washing in lieu of wiping.

The engineering team was onsite 5 days/week to operate the system and collect data. Because one of the main objectives was to identify maintenance requirements for various components, maintenance schedules were not developed ahead of time. Daily activities included checking for leaks, backing up of filters, and scaling on metal components (including electrodes). Daily water quality analysis was performed to monitor functionality, and included pH, conductivity, and oxidative reduction potential (ORP) in all tanks, and free chlorine and total chlorine in the process tank directly after electrochemical processing was complete. More detailed measurements including NH_3_, chemical oxygen demand (COD), and enumeration of bacteria and helminth eggs were done weekly when feasible by a lab at the local University, PSG Institute of Medical Sciences and Research. Samples were also collected and transported monthly to a third-party lab (T.S. Stanes, Coimbatore) for a comprehensive analysis of the water quality.

### 2.2 Prototype system and operation

The prototype system was based on one previously described in detail (Rogers et al., 2018) with the following modifications necessary to accommodate a 6-L flush volume (Fig. 1C): 1) an inlet tank (75 L) was added to buffer the larger flushes; 2) the volumes of the pre-process and holding tanks were increased to maintain residence times; 3) the capacity of the GAC1 columns was doubled by the addition of two columns. The electrochemical cell used was an off-the shelf system (Hayward Salt & Swim 3C) consisting of 13 dual-sided mixed metal oxide electrodes, 64 cm^2^ in area separated by 3-mm gaps. The system was equipped with 6 GAC columns in total, each 1982 mm in height and 128 mm in diameter made from PVC and custom-fabricated onsite. GAC was charcoal based (grade IV: 900, size 8–16 mm) and procured from Green Dust Bioprocess Engineer (Coimbatore). GK Controls Private Limited (Pune) manufactured the frame containing the GAC columns and all liquid processing tanks. Each tank was made using high-density polyethylene (HDPE).

The treatment system was automated by an industrial controls system based around the Siemens S7-1200 Programmable Logic Controller (PLC) system and operated by a touchscreen Human Machine Interface (HMI). The PLC received inputs from float switches located in the treatment system and controlled pumps through a series of relays. The process logic operated in automated or manual mode. In automated mode, the system continuously processed blackwater when there was enough liquid available for processing. In manual mode, treatment was initiated by the operator via the HMI; manual mode was used to collect data during processing.

A drain diverter was incorporated into the plumbing of the toilet which sent all flushed material to the septic tank onsite when activated. The drain diverter was programmed to activate in the case of power loss to the system or when the pre-process tank was full to prevent an overflow of liquid into the system. All tanks were equipped with overflow drains to the septic tank for additional safety.

During the preliminary phase of testing, the treated water in the holding tank was discharged to the septic tank until disinfection was verified over three consecutive measurements. Once disinfection was confirmed, the treated liquid was recycled into the flush cistern by manually switching a valve from open loop (treated liquid to septic tank) to closed loop (treated liquid recycled to flush cistern). The system only recycled flush liquid if the system was manually set to recycle and the holding tank low float switch was engaged, meaning there was enough liquid in the holding tank. Otherwise, the municipal tap water was used to fill the toilet cistern. When the loop was closed, the cistern pump automatically sent liquid from the holding tank to the cistern to refill the tank when a cistern flush was detected through a watertight microswitch (Honeywell, V15 W-DZ200A06-AW1) located inside the cistern and connected to the flush button.

Total system energy data were automatically recorded with an application called Kelv°n (Biomass Controls, available at https://apps.apple.com/us/app/kelvin-biomass-controls/id905043494 and https://play.google.com/store/apps/details?id=com.clearstak.kelvin&hl=en). The Intelligent Biomass Controller (IBC) processed and recorded signals every 5–50 s from a WattNode which transmits a signal based on the wattage being measured. The WattNode itself processed signals from a current transducer, which converted a measured amperage to an analog signal.

### 2.3 Analytical methods

Onsite measurements of conductivity, pH, and ORP were taken using a handheld Myron L Ultrameter 6PII. Free chlorine and total chlorine were measured using HACH - DPD Free Chlorine Reagent Powder Pillows and HACH – DPD Total Chlorine Reagent Powder Pillow Packs respectively, with a HACH DR 900 Colorimeter using program 87 Chlorine, F&T PP MR. Chlorine was analyzed halfway through and at the end of each electrochemical process when the system was operated manually. Ammonia was measured with Nitrogen-Ammonia Reagent Set Salicylate Method and the same colorimeter. COD was measured with HACH COD Digestion Vials – High Range vials and a HACH DRB 200 Digester Block was used for incubating samples before reading in the colorimeter. Turbidity was measured using the HACH 2100Q IS Portable Turbidimeter. Color was measured with a HACH CO-1 test kit.

Helminth egg enumeration was conducted according to a modified ambic method by isolation and microscopic evaluation as described in Grego et al. (2018). Bacteria were enumerated in the lab at PSG Institute of Medical Sciences and Research by the most probable number (MPN) method as previously described (Sellgren et al., 2017). Briefly, triplicate serial dilutions in lysogeny broth were prepared in sterile 48-well culture plates; the lysogeny broth used was a mixture of 10 g/L each of tryptone and NaCl and 5 g/L yeast extract in deionized water. Samples were incubated at 37 °C for 24 h before being analyzed according to the FDA method (Blodgett, 2010).

Monthly third-party testing of water quality parameters and microbial counts was performed according to the standard methods listed in SI-1.

## 3 Results and discussion

### 3.1 System performance: effluent quality

**Fig. 2:**
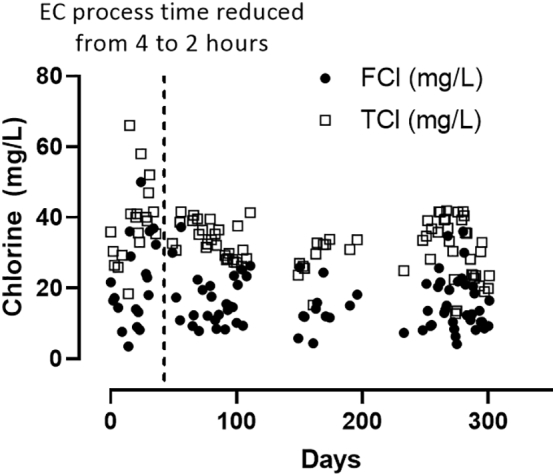
Chlorine production throughout the testing period. Data shown are individual free and total chlorine measurements taken in the process tank at the end of the electrochemical process (n = 94). On day 49 after commissioning, the electrochemical process was reduced from 4 to 2 h with no negative impact on chlorine production.

The highest priority in testing involving users is their health and safety; in the context of a non-sewered sanitation system designed for water reuse, this necessitates continuous monitoring of disinfection efficacy. However, because microbial enumeration is a costly and time-consuming process (requiring at least two days turnaround time in the field), these data could only be gathered once per week. For this reason, chlorine production was monitored daily as a proxy for biological safety. During the first 45 days of testing, chlorine production was consistently high, with free chlorine (FCl) averaging 47 mg/L and total chlorine (TCl) 64 mg/L. Beginning with the test on day 49, the electrochemical process time was reduced from 4 to 2 h and chlorine production remained sufficient with an average of 16 mg/L FCl and 32 mg/L TCl for the remainder of the study. No consistent reduction in chlorine production was observed throughout the entirety of the study (Fig. 2).

**Fig. 3:**
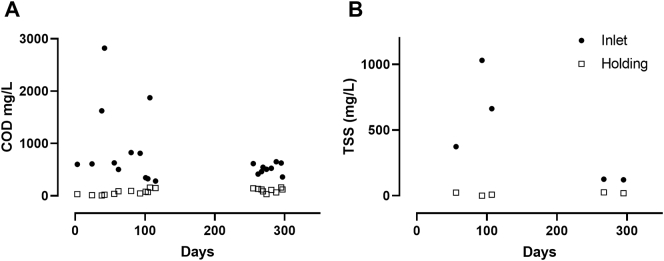
Inlet and holding tank measurements of COD (A) and TSS (B).

Effluent pH was within the range of 7.2–8.1 in all measurements (n = 98). Influent COD ranged from 282 to 2820 mg/L, and was reduced below 150 mg/L in 19 of 21 effluent COD measurements; of these measurements, seven were also below 50 mg/L (Fig. 3A). Similarly, total suspended solids (TSS) ranged from 374 to 1030 mg/L in the influent, and was reduced below 30 mg/L in all effluent measurements (n = 5) and below 10 mg/L in two effluent samples (Fig. 3B).

**Fig. 4:**
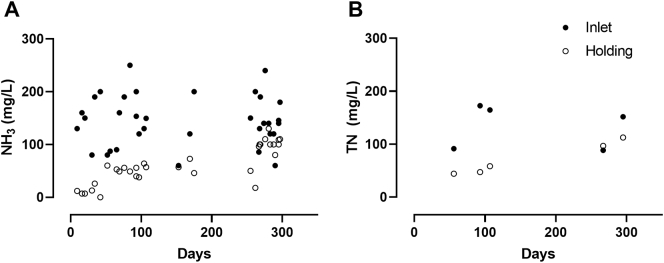
Inlet and holding tank measurements of NH_3_ (A) and total nitrogen (TN) (B).

**Fig. 5:**
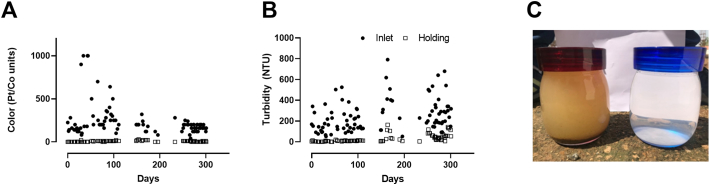
Color and turbidity removal. Data shown are individual color (A) and turbidity (B) measurements taken from the inlet and holding tanks. C: Inlet (left) and effluent (right) liquid samples show the clear, colorless liquid produced by the system.

Due to field testing constraints, more NH_3_ data points (Fig. 4A) were collected than TN (Fig. 4B). However, NH_3_ accounted for 84–99% of TN on days when both measurements were taken (n = 5 inlet and 5 outlet samples) suggesting that NH_3_ removal rates are a reasonable estimation of TN removal. The average NH_3_ removal rate was 58% (n = 34). Throughout the testing period the effluent color was always ≤30 Pt/Co units (Fig. 5A), and turbidity remained low with a slight increase in variability through the latter months of testing as discussed in Section 3.3.2 (Fig. 5B).

**Fig. 6:**
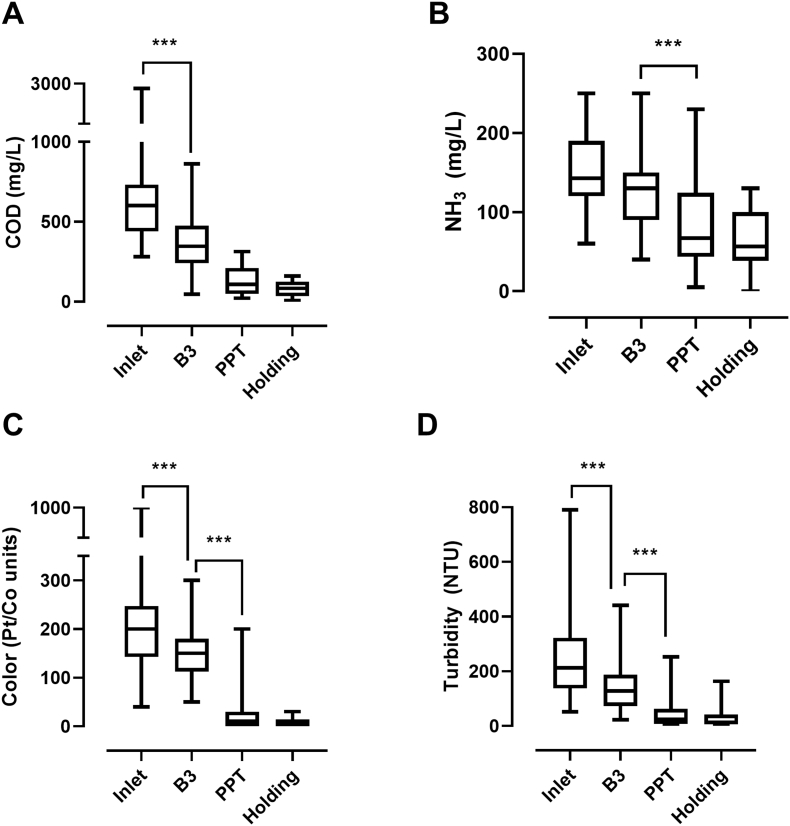
Water quality parameters at different stages of treatment. Data shown were collected over the course of the testing period; lines in the boxes represent medians, the boxes the 25th and 75th percentiles, and the error bars are minimum and maximum values; n = 21 (A), 34 (B), 96 (C), 94 (D). Comparisons among different stages were made with one-way ANOVA with a Sidak's multiple comparisons test. *** = p < 0.001 for the comparisons indicated. Statistical analysis was performed with GraphPad Prism v 8.0.1.

To determine the relative contributions of each stage of the liquid treatment process, water quality parameters were measured in different tanks (Fig. 6). A statistically significant removal of COD was observed between the inlet and third baffle (B3) tanks, indicating a substantial contribution of settling to COD removal in this system (Fig. 6A). Additional COD removal was observed between B3 and the PPT, although this difference was not quite statistically significant (p = 0.0723), likely due to the relatively low number of observations (n = 21) and wide variability in the inlet values. The majority of NH_3_ removal occurred between the B3 and PPT tanks, indicating removal primarily by GAC1 (Fig. 6B). Color and turbidity (Fig. 6C and D) were both significantly reduced between the inlet and B3 and between B3 and the PPT, indicating contributions from both settling and GAC1. No statistically significant differences were observed between the PPT and holding tank, indicating that the electrochemical process did not have a major effect on any of these parameters.

**Fig. 7:**
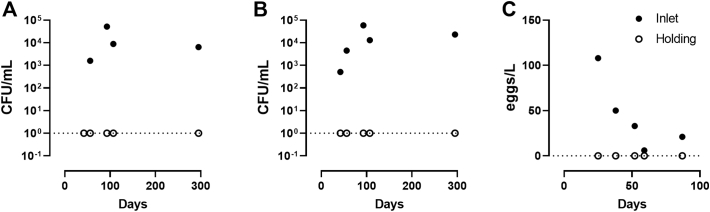
Pathogen removal. *E. coli* (A) and total coliform (B) measured in samples from the inlet and holding tanks. Dotted line indicates limit of detection for these assays (1 CFU/mL). C: Helminth eggs enumerated in samples from the inlet and holding tanks.

Influent *E. coli* and total coliforms ranged from 500 to 60,000 CFU/mL, and were reduced below the detection limit in all measurements (Fig. 7A and B). Similarly, the concentration of helminth eggs in the inlet tank varied from 6 to 108 eggs/L, and complete removal of helminth eggs was achieved in the system with zero counts in the holding tank (n = 5). Measurements across the stages of the treatment process revealed eggs in the settled sludge while none were found in the PPT, suggesting that settling was the major mechanism for removal in this system. As an additional safety measure for the effluent, a 10-micron nominal cartridge filter was installed between the process tank and the holding tank, though no viable helminth eggs were found to have been caught in this filter.

### 3.2 System performance: energy usage

Using data collected by the WattNode and IBC, an average instantaneous wattage was determined for operation of individual subcomponents of the system including background operations (pumps, etc.). Based on the electrochemical cell liquid processing batch size of 30 L and an average instantaneous wattage of the electrochemical process with background components of 100 watts, the entire system operates at 24 kJ/L. The required energy for the electrochemical cell to achieve disinfection was 11.5 kJ/L with a 24 VDC applied to the electrochemical cell for 2 h with an average current of 2 amps. It is possible the actual energy requirement for disinfection is less as the electrochemical process duration was 2 h throughout the entirety of testing and may have achieved disinfection with less time.

In lab studies of the system (Rogers et al., 2018), the energy requirement for disinfection to the detection limit in the electrochemical process was 32 kJ/L with only GAC1 filters and 20 kJ/L with GAC1 and GAC2 filters. During field testing, only 11.5 kJ/L energy was required for complete disinfection with only GAC1 filters in use.

It is important to note that blackwater during field testing in Coimbatore was more dilute than what was used in lab studies prior to field testing. The toilets in Coimbatore utilized a 6 L flush, while lab testing used 1.5 L. Furthermore, India is a washing culture, meaning that tap water used for anal cleansing entered the system with each use in addition to the cistern flush volume. The additional water entering the system resulted in an influent conductivity typically around 3 mS/cm, significantly less that the 15 mS/cm observed in lab studies (Hawkins et al., 2017; Rogers et al., 2018). The lower energy requirement for disinfection during field testing was likely due to a diluted influent blackwater when compared to the influent during lab testing, both because the energy required for electrochemical disinfection is known to increase with increased COD (López-Gálvez et al., 2012) and because the lower conductivity causes the electrochemical process to run at a lower current.

### 3.3 Component performance and maintenance required

Throughout the 10 months of operational field testing, 7860 L of blackwater was processed by the system. The electrochemical cell was able to produce sufficient chlorine to achieve disinfection throughout the entire testing period, and the GAC media never had to be replaced.

The solid-liquid separator required occasional maintenance as belts snapped. A second solid-liquid separator cassette with belts already installed was kept onsite as a spare and could be easily replaced in the case of multiple snapped belts. Onsite personnel would either replace the entire cassette, or replace the belts only on the installed cassette when multiple belts were broken. Frequency of solid-liquid separator maintenance can be found in SI-2. Though periodic replacement of belts was required, the solid-liquid separator did not require frequent cleaning to maintain functionality, as was found during the other field trial of this system in South Africa where toilet paper usage was common (Sahondo et al., 2019).

Sludge accumulation was measured in the inlet tank after 6.5 months of operation. The maximum depth in the tank was measured as 6 cm, from which the maximum total sludge volume accumulated in the 75 L tank was estimated to be 15 L after treating 5310 L of liquid, including liquid seen during the commissioning phase. The tank never had to be emptied of sludge throughout the duration of testing.

#### 3.3.1 Electrochemical cell

A minimum chloride concentration in the liquid is required to produce sufficient chlorine in the electrochemical process. It is expected that urine will provide sufficient salt for this process; however, large flush volumes may dilute the urine enough that addition of supplemental salt is required to ensure sufficient chlorine production (Sahondo et al., 2019). Because the water at this site was from a borewell (a very common solution in India) it contained a high mineral content (chloride concentrations measured between 200 and 460 mg/L); as a result, no salt addition was required at this site to achieve adequate chlorine production. For the same reason, the electrodes in the electrochemical cell began to scale over time, reducing chlorine production. The electrodes were cleaned periodically with a muriatic acid solution as detailed in SI-2; an interval of 75 h of operation between cleanings was found adequate to maintain cell functionality.

**Fig. 8:**
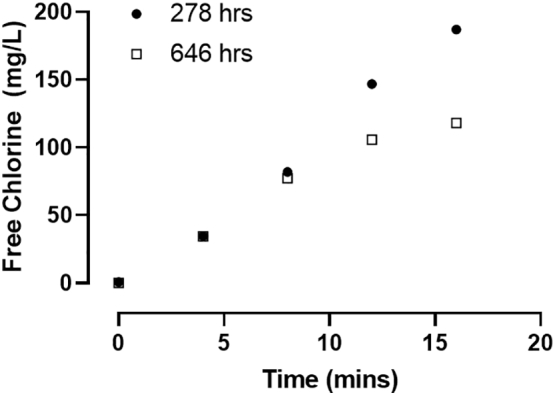
Chlorine evolution rate. Shown are free chlorine measurements taken at the times indicated with the electrochemical cell immersed in 20 mM NaCl and 24 V applied.

To assess the functionality of the electrochemical cell over the course of the testing period, chlorine evolution rate tests were conducted where the electrochemical cell was submersed in a tank with 8 L of water with 20 mM NaCL added. The chlorine produced was measured at timed intervals of 4 min to 16 min total. Results from this test show a 37% reduction in chlorine evolution rate following 646 h of operation compared to the previous test following 278 h (7.7 mg/L/min vs 12.1 mg/L/min respectively, Fig. 8). However, the electrodes produced sufficient levels of chlorine to achieve disinfection without any operational changes throughout the duration of field testing.

#### 3.3.2 GAC columns

Occasionally the GAC columns required backwashing when it was observed the columns were backing up with water. The GAC1 columns were backwashed 3 times, with an average interval of 1167 h of operation between backwashings, and were operational for a total of 3992 h. The GAC2 columns were backwashed one time but were only operational for 1908 h (see below).

**Fig. 9:**
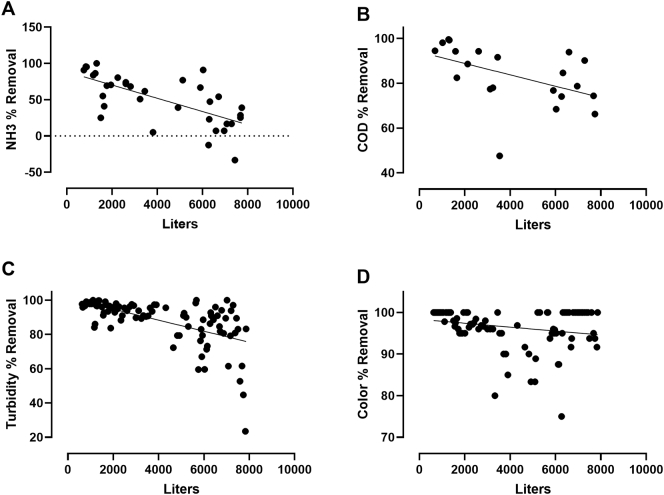
Removal rates due to GAC1 columns for NH_3_ (A), COD (B), turbidity (C), and color (D). Data were calculated based on measurements taken from the third baffle tank (B3) and PPT, and are plotted with respect to total liters blackwater treated by the system. Dotted lines indicate best-fit linear regressions; in all cases the slope was significantly non-zero, indicating diminished removal rates over the lifetime of the GAC.

Removal rates attributable to the GAC1 columns declined over time, as is expected with an adsorptive medium (Fig. 9). However, extrapolation of the COD removal rates observed (Fig. 9B) suggests that the system could treat approximately 17,300 L of blackwater (at the concentrations observed at this site) before the COD removal rate would fall below 50%. Given that the average liquid input into the system at this site was 7.5 L per toilet use (Welling et al., unpublished data), this would correspond to just over 2300 uses of the toilet before the GAC1 media would require replacement. The more dilute influent noted in Section 3.2 also likely contributed to the long service lifetime expected for the GAC columns based on these data. These findings largely corroborate and expand on the findings of the field trial of this system in South Africa, in which the GAC columns also performed well over many months, though in that case the system treated only ~1/3 of the total liquid volume over the course of the trial (Sahondo et al., 2019).

GAC2 was turned off for the majority of field testing because COD removal was high enough without it and having GAC2 on resulted in removal of all residual chlorine in the holding tank. During the last few months of the field testing period, COD removal rates began to decrease, so GAC2 was turned back on to provide additional treatment. As expected, it was observed that while GAC2 was on, all residual chlorine was depleted in the holding tank. During this period, the holding tank liquid also showed MPN values above the detection limit. The lysogeny broth method used to enumerate bacteria in these tests is not specific for any particular class of bacteria (though coliforms cannot be ruled out), so it is not possible to distinguish between regrowth or contamination by ambient microbiota based on these data, though either would be made more likely by a depletion of the chlorine residual by the GAC2 columns.

**Fig. 10:**
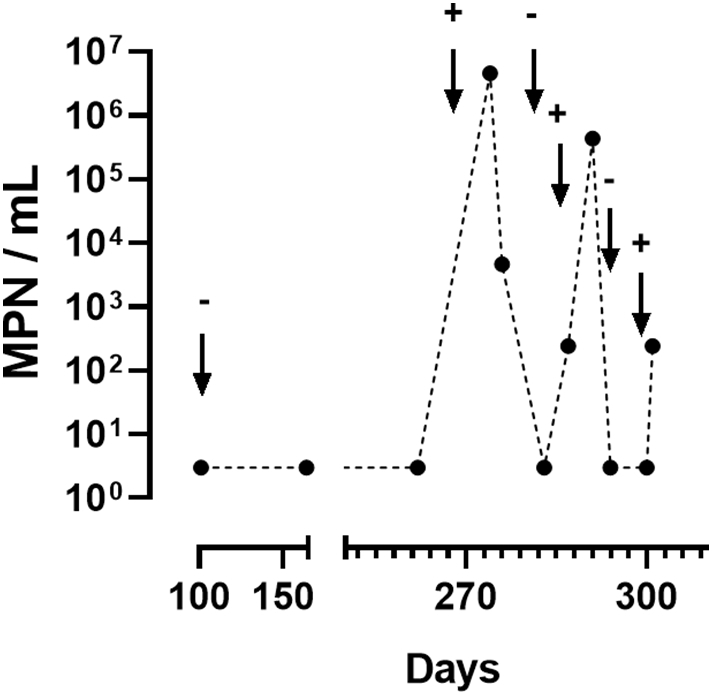
Bacterial counts (MPN) from the holding tank with and without GAC columns in operation. + indicates when the GAC2 columns were turned on, − indicates when they were turned off.

After the discovery that GAC2 removed all residual chlorine, the GAC2 columns were turned off. The holding tank was completely disinfected and the GAC2 columns were again turned on to test for recontamination. This sequence was performed 3 times, with the holding tank disinfected each time after samples were collected. Each test concluded GAC2 activation was consistently associated with contamination of the holding tank (Fig. 10).

### 3.4 System performance with respect to standards and user acceptability

System performance parameters are summarized in Table 1. During the 10 months of testing, average effluent COD and TSS values met the ISO 30500 Category B (restricted reuse) standards. The average TN removal rate of 65% fell just short of meeting the 70% removal rate required in the ISO 30500 standard. TP reduction did not meet the ISO standard of 80% removal; however, TP was always <16 mg/L throughout the entire system. *E. coli* in the effluent was always below the detection limit (1 CFU/mL) of the method used for analysis. The ISO 30500 standard requires a level of detection of 100 CFU/L or 0.1 CFU/mL, meaning a larger liquid sample would have been required for analysis to reach this level of detection. However, given the long residence time of liquid in the holding tank, *E. coli* regrowth would likely have been observed in the tank if viable *E. coli* were present following treatment. Effluent fecal coliforms were below the detection limit in all tests and met the Indian standard.

**Table 1:** Summary of effluent water quality and associated standards. Data are mean ± S.D. (range).

	ISO 30500^a^		India standard^b^	Influent	Effluent	% removal	n
	Category A	Category B					
TSS (mg/L)	≤10	≤30	<20	689 ± 268 (374–1030)	11 ± 10 (1–24)	98%	5
COD (mg/L)	≤50	≤150	<50	772 ± 638 (280–2860)	81 ± 48 (6–206)	90%	33
TN (mg/L)	70% removal		<10	143.0 ± 36.5 (91.5–172.8)	50.1 ± 6.1 (44.3–58.6)	65%	5
P (mg/L)	80% removal		<1	11.4 ± 4.3 (5.5–15.5)	8.6 ± 4.2 (3.5–13.7)	25%	5
pH	6–9		5.5–9	7.7 ± 0.2 (7.1–8.4)	7.7 ± 0.2 (7.2–8.1)	–	98
BOD (mg/L)	–		<20	243 ± 111 (142–406)	24 ± 17 (6–51)	90%	5
Fecal coliforms (MPN/100 mL)	–		<230		<200^c^		
*E. coli* (CFU or MPN/L)	<100		–		<1000^d^	–	5

a ISO/FDIA 30500: non-sewered sanitation systems—prefabricated integrated treatment units—general safety and performance requirements for design and testing, ISO, 2018.

b Green Tribunal Principal Bench, New Delhi, Original Application No. 1069/2018, Deshpande vs. Union of India and Ors., 30 April 2019.

c Limit of detection for this assay was 2/mL or 200/100 mL; all measurements were below the detection limit.

d Limit of detection for this assay was 1/mL or 1000/L; all measurements were below the detection limit.

Although there were no standard requirements for turbidity and color in the community where we tested at the time of testing, we know from informal conversations with the users that the appearance of the effluent is critical for user acceptability of reused wastewater. In this study, the effluent was consistently clear and colorless with removal rates of 88% and 97% for turbidity and color, respectively (Fig. 5), and after having met the biological disinfection requirements for three consecutive measurements, the holding tank was connected to the toilet cistern and the treated effluent used for flushing. The users of the toilet facility were informed verbally and with public notices that water reuse had been implemented. Operation with reuse was maintained for three weeks (SI-2) without any complaints logged from the users. This outcome suggests that reused blackwater treated to the quality that this prototype achieved could be acceptable for reuse in flushing.

## 4 Conclusions

The onsite blackwater treatment prototype deployed in Coimbatore, India successfully treated 7860 L of blackwater over a 10-month testing period, operating 24 h/day, 5 days/week, consistently producing treated water that was clear and colorless, met stringent disinfection thresholds for both *E. coli* and helminth eggs, and met or nearly met both local and recently adopted international discharge standards for non-sewered sanitation systems. Two key components (the activated carbon filters and the electrochemical cell) operated throughout the study without replacement and predictable maintenance, and the system never required de-sludging. Water reuse for toilet flushing was seamlessly implemented without affecting user perceptions or acceptance. The results of this study demonstrate the applicability of this approach to onsite blackwater treatment in the Indian use case. Next-generation treatment systems are currently under development that will be informed by these findings and improve upon this system in terms of performance, cost, and ease of maintenance.

## Declaration of competing interest

The authors declare that they have no known competing financial interests or personal relationships that could have appeared to influence the work reported in this paper.

## Appendix A Supplementary data

Supplementary material Image 1
