# Supplementary material for: Field testing of a household-scale onsite blackwater treatment system in Coimbatore, India
Source: Sci Total Environ. 2020 Apr 15;713:136706. doi: 10.1016/j.scitotenv.2020.136706 (PMC7043008; doi:10.1016/j.scitotenv.2020.136706)
Supplement: Supplementary file 1 — Supplementary material [file mmc1.docx]

**Supplementary Information**

**SI-1 Stanes third party lab water quality testing methods**

| **Parameter** | **Unit** | **Standard** |
| --- | --- | --- |
| Color | Hazen | IS 3025 (Part 4) 1983 |
| Turbidity | NTU | IS 3025 (Part 10) 1984 |
| Total Suspended Solids | mg/L | IS 3025 (Part 17) 1984 |
| Total Dissolved Solids | mg/L | IS 3025 (Part 16) 1984 |
| pH (direct) | -- | IS 3025 (Part 11) 1983 |
| Alkalinity (as CaCO3) | mg/L | IS 3025 (Part 23) 1986 |
| Conductivity | mS/cm | IS 3025 (Part 14) 2013 |
| Chloride (as Cl) | mg/L | IS 3025 (Part 32) 1988 |
| Calcium | mg/L | IS 3025 (Part 40) 1991 |
| Phosphorus | mg/L | IS 3025 (Part 31) 1988 |
| Nitrogen Ammonia | mg/L | IS 3025 (Part 34) 1988 |
| Nitrate Nitrogen (as NO3) | mg/L | IS 3025 (Part 34) 1988 |
| Nitrite Nitrogen (as NO2) | mg/L | IS 3025 (Part 34) 1988 |
| Total Kjeldahl Nitrogen | mg/L | IS 3025 (Part 34) 1988 |
| Total Nitrogen | mg/L | IS 3025 (Part 34) 1988 |
| Chemical Oxygen Demand | mg/L | IS 3025 (Part 58) 2006 |
| Biological Oxygen Demand | mg/L | IS 3025 (Part 44) 1993 |
| E. Coli (colony count) | CFU/ml | IS 5887 P1 : 1976 |
| Coliforms (colony count) | CFU/ml | IS 5401 P1 : 2012 |
| E. Coli (MPN) | MPN/100 ml | IS 1622 : 1981 |
| Coliforms (MPN) | MPN/100 ml | IS 1622 : 1981 |

**SI-2 Timeline of Field Testing, Subcomponent Operation, and Maintenance**


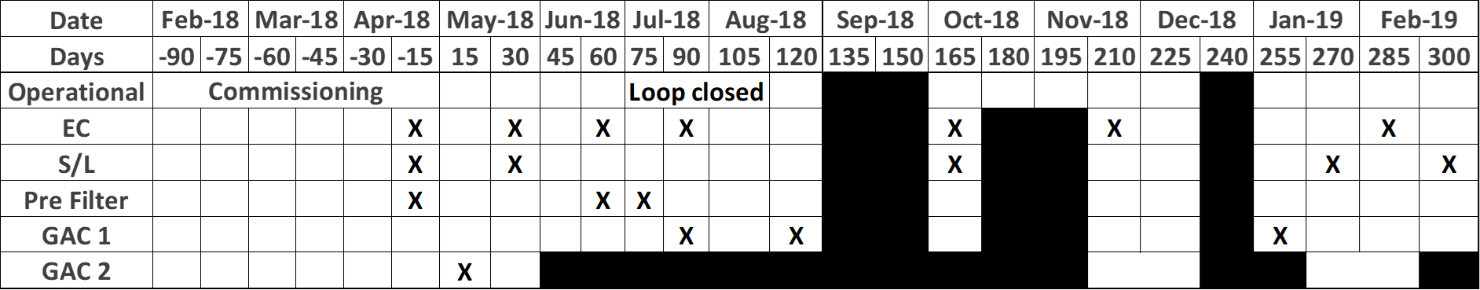


Black cells indicate subcomponent or system was off

“X” indicates maintenance was performed on subcomponent

Definitions of maintenance for each subcomponent:

1. EC = cleaning of electrochemical cell with muriatic acid
2. S/L = replacing belts of solid-liquid separator
3. Pre filter = cleaning debris on pre filter with tap water
4. GAC 1 = backwashing GAC 1 column with tap water
5. GAC 2 = backwashing GAC 2 column with tap water
